# The ZIP5 Ectodomain Co-Localizes with PrP and May Acquire a PrP-Like Fold That Assembles into a Dimer

**DOI:** 10.1371/journal.pone.0072446

**Published:** 2013-09-06

**Authors:** Cosmin L. Pocanschi, Sepehr Ehsani, Mohadeseh Mehrabian, Holger Wille, William Reginold, William S. Trimble, Hansen Wang, Adelinda Yee, Cheryl H. Arrowsmith, Zoltán Bozóky, Lewis E. Kay, Julie D. Forman-Kay, James M. Rini, Gerold Schmitt-Ulms

**Affiliations:** 1 Tanz Centre for Research in Neurodegenerative Diseases, University of Toronto, Toronto, Ontario, Canada; 2 Department of Laboratory Medicine and Pathobiology, University of Toronto, Toronto, Ontario, Canada; 3 Department of Biochemistry and Centre for Prions and Protein Folding Diseases, University of Alberta, Edmonton, Alberta, Canada; 4 Program in Cell Biology, Hospital for Sick Children, Toronto, Ontario, Canada; 5 Department of Biochemistry, University of Toronto, Toronto, Ontario, Canada; 6 Structural Genomics Consortium, Toronto, Ontario, Canada; 7 Molecular Structure and Function Program, Hospital for Sick Children, Toronto, Ontario, Canada; 8 Department of Molecular Genetics, University of Toronto, Toronto, Ontario, Canada; University of Maryland School of Medicine, United States of America

## Abstract

The cellular prion protein (PrP^C^) was recently observed to co-purify with members of the LIV-1 subfamily of ZIP zinc transporters (LZTs), precipitating the surprising discovery that the prion gene family descended from an ancestral LZT gene. Here, we compared the subcellular distribution and biophysical characteristics of LZTs and their PrP-like ectodomains. When expressed in neuroblastoma cells, the ZIP5 member of the LZT subfamily was observed to be largely directed to the same subcellular locations as PrP^C^ and both proteins were seen to be endocytosed through vesicles decorated with the Rab5 marker protein. When recombinantly expressed, the PrP-like domain of ZIP5 could be obtained with yields and levels of purity sufficient for structural analyses but it tended to aggregate, thereby precluding attempts to study its structure. These obstacles were overcome by moving to a mammalian cell expression system. The subsequent biophysical characterization of a homogeneous preparation of the ZIP5 PrP-like ectodomain shows that this protein acquires a dimeric, largely globular fold with an α-helical content similar to that of mammalian PrP^C^. The use of a mammalian cell expression system also allowed for the expression and purification of stable preparations of *Takifugu rubripes* PrP-1, thereby overcoming a key hindrance to high-resolution work on a fish PrP^C^.

## Introduction

Prion disorders are invariably fatal diseases of humans and animals [1]. In prion diseases, the host-encoded cellular prion protein (PrP^C^) undergoes a conformational transition to a disease-associated ‘scrapie’ conformer, commonly referred to as PrP^Sc^ [2]. In search of physiological interactors of PrP^C^, we recently observed that two members of the family of ZIP zinc transporters [3], ZIP6 (Slc39a6) and ZIP10 (Slc39a10), co-purified with PrP^C^ [4] and, subsequently, we discovered that the prion gene family descended in evolution from an ancestral ZIP gene [5]. ZIP zinc transporters are encoded by the *slc39a* gene family that consists of fourteen genes in humans and mice. ZIP6 and ZIP10, together with their phylogenetically closest paralog ZIP5 (Slc39a5), are members of the LIV-1 subfamily of ZIP zinc transporters (LZTs) that are characterized by a conserved sequence motif found in transmembrane domain V [6] and the presence of N-terminal ectodomains absent in non-LZT members of this protein family. The mechanism of evolution of the PrP founder gene from an ancestral LZT gene was most likely based on a retrotransposition event that led to a loss of sequences coding for the C-terminal multi-spanning transmembrane domain present in LZTs [7]. Consequently, the similarity between PrP and LZT sequences is restricted to the N-terminal ectodomains of LZTs. In addition to the overall similar sequence organization, these LZT ectodomains are predicted to share with PrP its lumenal/extracellular orientation and proximity to the membrane. Pair-wise alignments indicate that, in particular, LZT and PrP sequences found in fish genomes have retained considerable sequence similarity (up to 41%) [5].

The similarities between PrP^C^ and LZTs may also extend to the physiological function of these proteins. It has been known for some time that ZIP6-deficient zebrafish embryos fail to undergo an essential morphogenetic arrangement that occurs during development at the gastrula stage [8]. More recently, it was found that zebrafish deficient for PrP-1, one of three PrP-like genes encoded by the *Danio rerio* genome, are characterized by a strikingly similar developmental arrest phenotype [9]. Moreover, mammalian PrP introduced into PrP-1-deficient zebrafish embryos was shown to partially rescue the developmental arrest phenotype caused by the absence of PrP-1 [9], [10], and fish infected with prions were shown to develop signs of neurodegeneration and proteinase K-resistant amyloid-like deposits that stained with antibodies against PrP [11].

The presence of ZIPs 6 and 10 in co-immunoprecipitates (co-IPs) of PrP^C^ suggested that these proteins may, at least partially, reside in proximity to PrP^C^ [5]. Currently lacking, however, is information on their subcellular localization relative to PrP^C^. Even less well characterized is the relationship, if any, between PrP^C^ and ZIP5, the third member of a subbranch of LZTs that is most closely evolutionarily related to PrP. Quantitative RT-PCR analyses of transcripts in neuroblastoma cells (N2a) established that ZIP5 does not naturally occur in these cells [12], thereby providing an explanation for why, in contrast to ZIPs 6 and 10, this particular LZT was not detected in the original PrP^C^ co-IP studies.

High-resolution structural data are available for wild-type and mutant PrP^C^ from a wide range of organisms including humans, chicken, turtle and wallaby [13]. These investigations show that PrP^C^ possesses a bipartite structure consisting of a largely disordered N-terminus and a globular C-terminal domain comprised of three α-helices and a short anti-parallel β-sheet [14]. No high-resolution structure of a ZIP metal ion transporter has been reported. Both PrP^C^ and the above LZTs share with an overall small number of proteins the ability to simultaneously bind multiple divalent metal ions through histidine-containing motifs embedded in N-terminal repeat sequences [6], [15]. The high-resolution structure of a fish PrP^C^ or the PrP-like ectodomain of a relevant LZT would certainly be valuable. A comparison with the mammalian PrP^C^ structure would not only indicate the extent to which members within the PrP protein family have structurally diverged, but it may also reveal shared structural features common to PrPs and LZT ectodomains that might be functionally important. Previous attempts to obtain a high-resolution structure for a fish genome-encoded PrP have been unsuccessful [16]. Specifically, the bacterial expression systems used to produce material for the NMR analysis of mammalian and other tetrapod PrPs failed to produce suitable samples when used for *Takifugu rubripes* (pufferfish) PrP-1 (Tr_PrP-1) production [17]. Plausible scenarios suggesting why these earlier attempts were frustrated are many and include the possibility that fish PrP requires native posttranslational modifications or other cellular co-factors for folding.

The current study shows that PrP^C^ is biochemically more similar to ZIP5 than to ZIPs 6 or 10. When expressed in neuroblastoma cells, ZIP5 is directed to largely identical molecular environments as PrP^C^ and both proteins are endocytosed through Rab5-decorated vesicles. Structural threading analyses predict that pufferfish PrP-1 and the ectodomain of mouse ZIP5 acquire folds similar to mammalian PrP^C^. Difficulties in obtaining recombinant ZIP5 suitable for structural analyses were overcome by moving to a mammalian cell expression system. A method is presented that allowed the expression and purification of stable preparations of the PrP-like ZIP5 ectodomain, and subsequent analyses showed that the protein is dimeric and possesses a largely globular fold with an α-helical content similar to PrP^C^. A comparison of the PrP-like ZIP5 ectodomain with pufferfish PrP-1, purified by the same methodology, revealed that both proteins share biophysical characteristics that are similar, but not identical, to mammalian PrP^C^.

## Materials and Methods

### Clones

For recombinant expression, murine ZIP5 (GenBank accession number EDL24562.1, residues 20-207) with an N-terminal 10x poly-histidine (pHis) tag, followed by a three amino acid linker sequence (amino acids ‘SSG’) and a thrombin cleavage site was cloned into the pET-19b vector (Novagen, Madison, WI, USA) and transformed into *E. coli*. In expression vectors designed for the purification of the PrP-like ZIP5 ectodomain, a non-conserved cysteine residue at amino acid position 183 was mutated to a serine (‘CPALLY’ to ‘SPALLY). Notably, some closely related ZIP proteins (e.g., human ZIPs 4 and 12) naturally possess a serine residue at the corresponding position. The same murine ZIP5 or pufferfish PrP-1 (GenBank accession number AAN38988, residues 301–424) sequences were expressed in (HEK-293S) GnTĪ cells (deficient in *N*-acetylglucosaminyltransferase I) [18] using the recently described inducible *piggyBac* transposase-based mammalian cell expression system [19]. In this case, the molecules were expressed as fusion proteins containing an N-terminal Protein A purification tag followed by a fifteen-amino-acid linker sequence (amino acids ‘GNSGSGSSGGSGSGG’), the TEV recognition site (‘ENLYFQ’) and finally the target protein of interest. The Rab5-GFP expression product codes for the small GTPase Rab5 fused to a C-terminal green fluorescent protein (GFP) [20], [21]. All plasmids were sequence-verified.

### Antibodies

The mouse monoclonal anti-HA.11 antibody was obtained from Covance (cat. no. MMS-101P; Covance, Princeton, NJ, USA) and the mouse monoclonal anti-FLAG M2 antibody from Sigma-Aldrich (cat. no. F3165; Sigma-Aldrich, Oakville, ON, Canada). For the detection of PrP, we used mouse monoclonal anti-PrP Sha31 (cat. no. A03213; Medicorp, Montreal, QC, Canada) and rabbit monoclonal anti-PrP (cat. no. 2063–1; Epitomics, Burlingame, CA, USA). For the generation of polyclonal antibodies, peptides derived from mouse ZIP5 (‘CRLGHHEPPTGRAA’), ZIP6 (‘CGTRFVETIETPK’) and ZIP10 (‘CNHDHSEQYEHNR’) proteins were synthesized, conjugated to KLH and injected into Charles River Laboratories SPF rabbits (Division of Comparative Medicine, University of Toronto). Upon exsanguination, antibodies were purified from the rabbit sera by affinity chromatography on matrices generated by immobilizing the aforementioned antigenic peptides with the SulfoLink Immobilization Kit for Peptides (cat. no. 44999; Pierce, Rockford, IL, USA) according to the manufacturer's instructions. The secondary antibodies used were anti-mouse (cat. no. 170–6516; Bio-Rad, Mississauga, ON, Canada) or anti-rabbit (cat. no. 170–6515; Bio-Rad).

### Structural threading

The sequences of mouse ZIP5 (Mm_ZIP5; residues 96–212) and *Takifugu rubripes* PrP-1 (Tr_PrP-1; residues 28–450) were submitted to the Fold and Function Assignment System (FFAS) server [22] for automated structure prediction and structural threading. A number of alternatively truncated sequences were submitted to the FFAS server in order to minimize threading artifacts based on incorrectly chosen domain boundaries. The FFAS server produced a ranked list of structural threading models built on a profile-based sequence alignment and fold recognition algorithm that compared the targeted sequences to all entries in the Protein Data Bank (PDB). All list entries that exceeded the significance threshold of −9.5 came from published structures of PrP^C^. The known structure of *Xenopus laevis* PrP (Xl_PrP) (16) was chosen as a template for both the Tr_PrP-1 and Mm_ZIP5 models to make a direct comparison of these models more meaningful. The DaliLite server [23] was used to compare the Tr_PrP-1 and Mm_ZIP5 models with the Xl_PrP structure. The graphical representations were generated using the Chimera package [24].

### Confocal microscopy and co-immunofluorescence analysis

Confocal microscopy analyses of mouse neuroblastoma (N2a) cells were performed as previously described [12]. Briefly, the image acquisition was controlled by Volocity software (PerkinElmer, Waltham, MA, USA), and following automatic assignment of detection thresholds, regions of interest were manually drawn around cells and co-localization coefficients for individual channels were calculated from 20–30 layers of individual cells. Three technical replicates were performed. Data were plotted as mean percentage overlaps with standard error. Levels of divalent cations present in the cell culture medium (MEM +10% FBS) had been measured by inductively coupled plasma atomic emission spectroscopy (ICP-AES) previously and were determined as follows: Zn^2+^, 3.3 μM; Fe^2+^, 2.6 μM; Cu^2+^, 0.3 μM [12].

### Glycosylation analysis

Upon N2a cell lysis, 20 μg protein samples were denatured in 0.5% SDS and 1% β-mercaptoethanol at 99°C for 10 min, as previously described [12]. Sodium phosphate and NP-40 were added to final concentrations of 50 mM and 1%, respectively. 5 μL (2,500 units) of PNGase F (New England Biolabs, Ipswich, MA, USA) was added and the mixture incubated at 37°C overnight. See ‘Mammalian expression and protein purification’ section for details on endo-β-N-acetylglucosaminidase A treatment.

### Recombinant expression and protein purification

pHis-ZIP5-pET-19b was expressed in the RIL (DE3) Arctic Express strain (which contains extra copies of the *argU*, *ileY,* and *leuW* tRNA genes) (Agilent Technologies, Santa Clara, CA, USA). These cells co-express the cold-adapted chaperonins Cpn10 and Cpn60 from the bacterium *Oleispira antarctica* and have been shown to increase the yield of soluble proteins [25]. pHis-ZIP5 was essentially purified as described previously [26] with minor modifications. Briefly, the cell culture was started by inoculating 100 μL of a glycerol stock of transformed cells into 50 mL LB medium supplemented with 50 μg/mL carbenicillin in a 250 mL Erlenmeyer flask. The culture was incubated overnight at 37°C on an Innova 4000 incubator shaker platform (New Brunswick Scientific, Edison, NJ, USA) rotating at 250 rpm. Bacteria were pelleted at 4000×*g* at 4°C for 15 min in an Avanti J-26XP centrifuge equipped with a JA-12 rotor (Beckman Coulter, Indianapolis, IN, USA). Next, cells were resuspended in 1 L of M9 minimal media containing 0.5 g/L ^15^NH_4_Cl and 50 μg/mL carbenicillin in a 2.8 L Erlenmeyer flask and grown for 3 h at 30°C on an Innova 44 incubator shaker platform (New Brunswick Scientific), rotating at 250 rpm, to an OD_600_ of 0.6. The temperature was then set to 11°C, the culture adjusted to the new temperature for 30 min and ZIP5 expression induced in the presence of 1 mM IPTG for 16 h. Cells were pelleted as described above but using a JLA 9.1000 rotor at 7000 rpm, resuspended in Lysis Buffer (20 mL of 20 mM Tris pH 8.0, 30 mM imidazole) and then sonicated on ice for 5 min using a Vibra Cell sonicator (Sonic and Materials, Danbury, CT, USA) equipped with a large tip and pulsed at 50% for 300 s, output control ≤5. Lysed cells were ultracentrifuged in a Beckman Coulter Optima L-90 K ultracentrifuge in a 70Ti rotor for 1 h at 45,000 rpm and 4°C. Native pHis-ZIP5 was purified from the ultracentrifugation supernatant using **Strategy 1** or refolded from insoluble inclusion bodies using **Strategy 2**.

For **Strategy 1**, the supernatant was loaded onto a 5 mL nickel-nitrilotriacetic acid (Ni-NTA) resin column pre-equilibrated in Lysis Buffer. After extensive washing by passing 10 column volumes of Lysis Buffer through the matrix, native pHis-ZIP5 was eluted in 10 mL of 20 mM Tris pH 8.0, 300 mM imidazole.

For **Strategy 2**, the pellet containing the pHis-ZIP5 inclusion bodies was washed in 20 mL of Lysis Buffer and ultracentrifuged as described above. The inclusion bodies were solubilized overnight in 20 mL Solubilization Buffer (20 mM Tris, pH 8.0, 6 M guanidine hydrochloride (GdnHCl), 100 mM HNa_2_PO_4_, 10 mM reduced glutathione, pH 8.0) under gentle rocking at room temperature. Next, the resuspended mixture was ultracentrifuged and the supernatant added drop-wise and under continuous stirring to 20 mL of Ni-NTA resin that had been pre-equilibrated in Solubilization Buffer. The protein-resin mixture was then poured into an empty column (2 cm diameter and 20 cm height) and washed with 80 mL of Solubilization Buffer. To refold ZIP5 bound on the Ni-NTA beads, a 200 mL linear gradient of Solubilization Buffer → Refolding Buffer (20 mM Tris, 100 mM HNa_2_PO_4_, pH 8.0) was passed through the matrix with the aid of a GM-1 Gradient Mixer (Amersham, Uppsala, Sweden). To remove weak and non-specific binders to the affinity matrix, the Ni-NTA column was subsequently washed with 80 mL of Refolding Buffer containing 50 mM imidazole. Finally, pHis-ZIP5 was eluted with 40 mL of Refolding Buffer containing 500 mM imidazole. Purified refolded pHis-ZIP5 was dialyzed against 4 L of 20 mM Tris pH 7.5, 150 mM NaCl for 6 h at 4°C, replacing the buffer every 2 h. pHis-ZIP5 was concentrated using Amicon Ultracel 10 K concentrators (Millipore, Billerica, MA, USA) in a Sorvall Legend RT centrifuge at 4000 rpm. The protein yield was 0.1 mg/L of media for native pHis-ZIP5 and 5 mg/L for refolded pHis-ZIP5 from inclusion bodies, respectively.

### Mammalian expression and protein purification

The stably transfected (HEK-293S) GnTĪ bulk cell cultures were grown at 37°C, 5% CO_2_, in 250 mL of DMEM/F-12 supplemented with 3% FBS (Invitrogen, Carlsbad, CA, USA), 1X penicillin-streptomycin (Invitrogen), 1 mg/L of doxycycline (Sigma-Aldrich) and 1 mg/L of aprotinin (BioShop Canada, Burlington, ON, Canada) in 2 L roller bottles. Media containing the secreted Protein A (PA)-ZIP5 fusion protein was harvested every 72 h and replaced by fresh media. The pooled collected medium was concentrated 20-fold and the fusion protein was purified by IgG-Sepharose (GE Healthcare, Buckinghamshire, UK) affinity chromatography. The Protein A tag was removed by on-column tobacco etch virus (TEV) protease digestion. Eluted ZIP5 was treated with endo-â-*N*-acetylglucosaminidase A [27] in 50 mM Tris, 50 mM NaCl, pH 7.5, at 4°C for 24 h. The deglycosylated ZIP5 was then purified by Q Sepharose ion exchange chromatography and Superdex 200 gel filtration chromatography in 20 mM Tris, 20 mM NaCl, pH 7.5, and concentrated to 20 mg/mL.

### CD spectroscopy

All CD spectroscopy measurements were performed on a Jasco J-810 spectropolarimeter. CD spectra of 7.8 μM Mm_ZIP5 or Tr_PrP-1 in 20 mM Tris, pH 7.5, 150 mM NaCl were recorded between 190 nm and 250 nm in a 1-mm path length cuvette at room temperature. Ten scans were accumulated and averaged, with a response time of 4 s, a bandwidth of 1 nm, and a scan speed of 50 nm/min. Background spectra without protein were subtracted. The recorded CD spectra were normalized by conversion to mean residue molar ellipticity [Θ] with units converted to degrees cm^2^ dmol^−1^. In urea unfolding experiments, 7.8 μM Mm_ZIP5 or Tr_PrP-1 were equilibrated at room temperature for 5 h in 150 μL of 20 mM Tris, pH 7.5, 150 mM NaCl at urea concentrations ranging from 0 M to 8.8 M in 0.4 M increments. Urea denaturation curves were recorded at 220 nm.

### Analytical ultracentrifugation

Sedimentation equilibrium experiments were performed in an An-60 Ti rotor in a Beckman Coulter Optima XL-A analytical ultracentrifuge. The >95% pure preparation of Mm_ZIP5 was dialyzed against 20 mM Tris pH 7.5, 150 mM NaCl. The protein concentration of this preparation before loading into the analytical ultracentrifugation cell (with a path length of 4 mm, volume of 130 μL and column height of 12 mm) was 1 mg/mL. As computed by the SEDNTERP program, the partial specific volume of the protein sample (V-bar) was 0.7273. Equilibrium distributions were analyzed after 24 h of centrifugation at 18,000 and 20,000 rpm at 4°C. The absorbance was recorded at 280 nm and data were analyzed using the Origin MicroCal XL-A/CL-I software package (version 4.0). Data measured at 18,000 rpm provided the best fit to a single-species model with a deduced average molecular weight of ∼40 kDa (calculated at 40,223 Da). This observed mass matched the expected mass for a dimeric assembly of ZIP5, giving rise to a ratio of average measured mass to theoretical monomer mass (20,209.07 Da) of ∼2 to 1.

### NMR analyses

NMR spectra of Mm_ZIP5 in 20 mM Tris, pH 7.5, 150 mM NaCl at 10 mg/mL were recorded at 37°C on a Varian Inova 800-MHz spectrometer equipped with a triple-resonance probe. ^15^N-^1^H HSQC spectra were collected as 1634×128 complex matrices with sweep widths of 12,775 and 1,620 Hz in the ^1^H and ^15^N dimensions, respectively. The ^13^C-^1^H HMQC spectrum was acquired with 168 transients using ^13^C natural abundance and 1536×100 complex matrices with sweep widths of 12,000 and 4,000 Hz in the ^1^H and ^13^C dimensions, respectively. All recorded data were processed using NMRPipe [28] and analyzed using Sparky (http://www.cgl.ucsf.edu/home/sparky/).

## Results

### ZIP5 exceeds ZIP10 in its degree of co-localization with PrP^C^ in neuroblastoma cells

**Figure 1:**
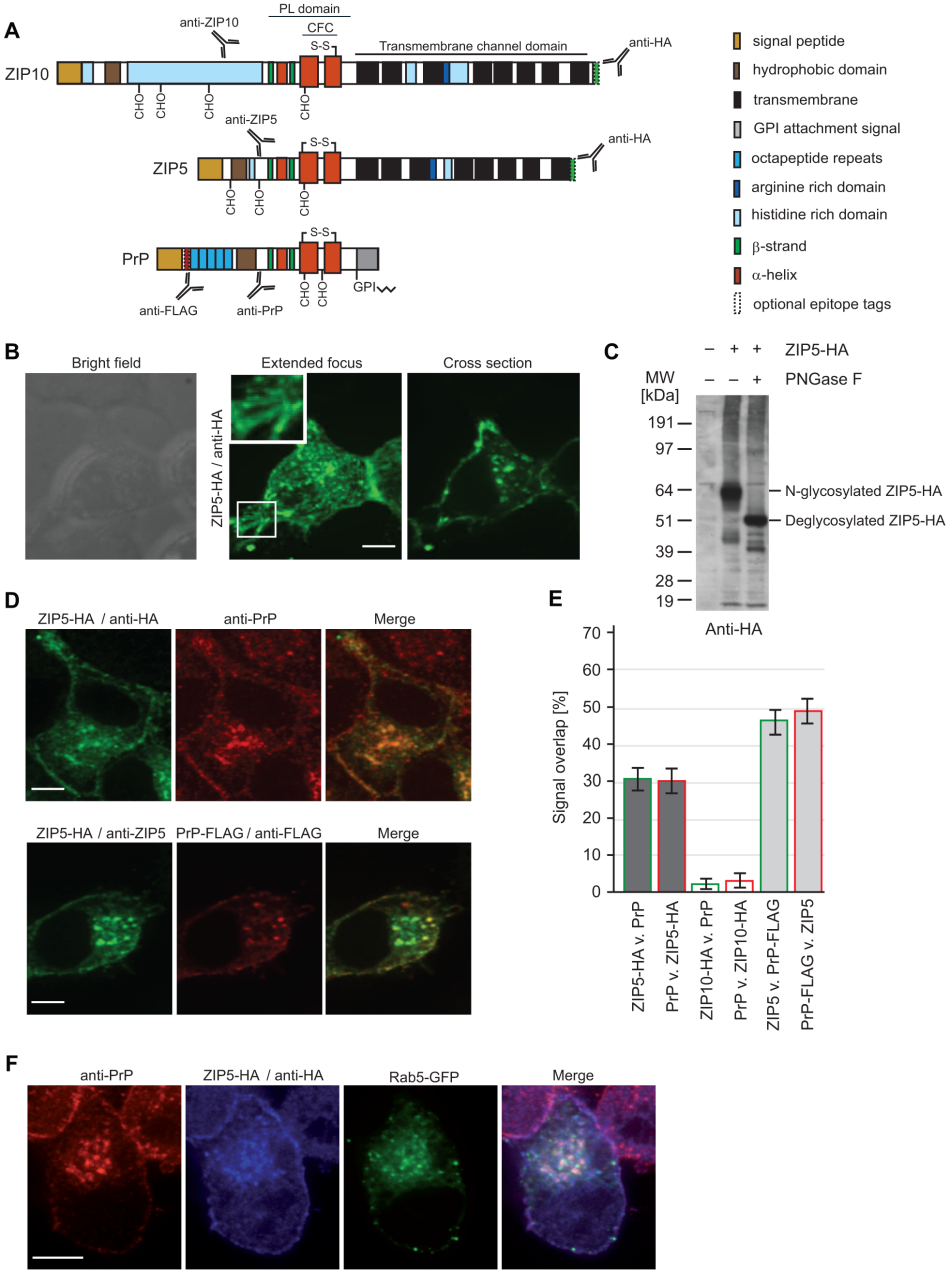
Co-localization of PrP^C^ and ZIP5 at the plasma membrane and in Rab5 immunoreactive endosomes. (**A**) Schematic comparing molecular features of ZIPs 5 and 10 with PrP^C^. (**B**) N2a cells were transfected with ZIP5-HA or ZIP10-HA (not shown) and the distribution of these heterologously expressed proteins analyzed by immunofluorescence. A comparison of bright field, confocal and extended focus views revealed predominant localization of ZIP5 at the plasma membrane and on vesicular structures with a negligible background staining in non-transfected cells. Both proteins can also be found in punctate structures within neuritic membrane protrusions (see insets). (**C**) ZIP5 is N-glycosylated within its ectodomain at multiple sites. (**D**) Representative co-immunofluorescence data generated with antibodies directed against endogenous PrP^C^ and the C-terminal HA-tag present on heterologously expressed ZIP5-HA. Of note is the prominent overlap of PrP^C^ and ZIP5-derived immunofluorescence signals; importantly, the spatial overlap of ZIP5- and PrP-dependent signals is preserved in cells which express both ZIP5-HA and PrP-FLAG when the detection is based on a polyclonal anti-ZIP5 antibody and a monoclonal anti-FLAG antibody. (**E**) Quantification of spatial overlap of fluorescence signals derived from PrP or PrP-FLAG with ZIP5-HA or ZIP10-HA. Split Mander's co-localization coefficients for comparing spatial signal overlaps of ZIPs and PrP were calculated in Volocity from at least 20 flattened z-stacks of individual cells. Data document mean percentage overlaps with standard error bars. (**F**) Co-immunofluorescence analysis of ZIP5 and PrP^C^ with the endosomal reporter protein Rab5. This analysis was based on a previously validated Rab5-GFP expression methodology. Please note the robust co-localization of both PrP^C^ and ZIP5 with early-endosome vesicles marked by the presence of Rab5. Scale bars are 25 µm.

Prior to this work, data by us and others suggested that ZIPs 5, 6 and 10, members of the subbranch of LZTs that are most closely related to PrP, predominantly localize to the cell surface [12], [29], [30]. A comparative analysis of the subcellular localization of these LZTs relative to PrP, however, was lacking. To minimize the risk that off-target binding might confound data interpretation when comparing the localization of the three LZTs with protein-specific antibodies by confocal immunofluorescence microscopy, a C-terminal HA tag was used for detection after transient transfection in N2a cells (**Figure 1A**). ZIP5 and ZIP10 gave rise to distinct puncta at the plasma membrane. Although additional intracellular puncta were evident, no nuclear signals were observed for ZIP5 and ZIP10, an observation consistent with the fact that these proteins traffic through the secretory pathway and are eventually degraded in endolysosomal compartments (**Figure 1B**) (see also [12]). In contrast, ZIP6 was primarily associated with perinuclear and intracellular punctate structures (not shown) and was not observed at the plasma membrane and, therefore, excluded from subsequent analyses.

In prior work, we had determined that ZIP10 is N-glycosylated at up to four N-glycan acceptor sites *in vivo* [12]. Overexpression of ZIP5 in N2a neuroblastoma cells followed by treatment with PNGase F and immunoblot analyses established that ZIP5 can also be N-glycosylated (**Figure 1C**), including at a highly conserved N-glycan acceptor site also known to be occupied in PrP and ZIP10 (not shown) [12].

Quantitative co-immunofluorescence analyses of endogenous PrP^C^ and HA-tagged ZIP5 or ZIP10 was also undertaken. Surprisingly, the degree of co-localization with PrP^C^ differed considerably between the two LZTs, with ZIP5-HA and ZIP10-HA exhibiting high and low levels of co-localization with PrP^C^, respectively (**Figure 1D**). The prominent co-localization of ZIP5 and PrP^C^ appeared to be robust and could be observed when the expression of the latter was driven from a plasmid coding for a FLAG-tagged variant of PrP^C^, using monoclonal anti-FLAG and polyclonal anti-ZIP5 antibodies for detection (**Figure 1E**). Finally, poor co-localization of ZIP5-HA or PrP-FLAG was observed with the early-endosome marker transferrin (not shown), but significant co-localization of both PrP and ZIP5-HA was observed with the early endosome reporter Rab5 (**Figure 1F**). Taken together, these experiments suggest that ZIP5 and ZIP10 share a predominant localization with PrP^C^ at the plasma membrane. In particular, ZIP5 strongly co-localizes with PrP^C^ and like PrP^C^ appears to utilize a vesicle subtype marked by the small GTPase Rab5 for its entry into the endolysosomal degradation pathway [31].

### PrP, ZIP5 and ZIP10 share key characteristics in their amino acid sequences and are predicted to acquire similar folds

**Figure 2:**
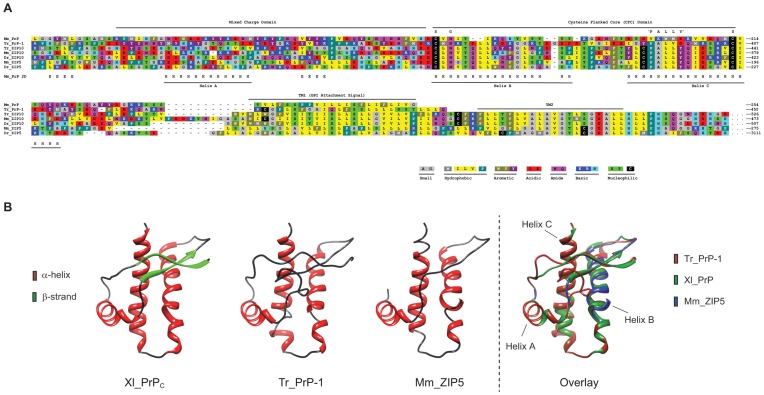
PrP-like ectodomain of mouse ZIP5 and pufferfish PrP-1 are predicted to acquire a PrP-like fold. (**A**) Multiple sequence alignment of selected prion protein sequences with ZIP5 and ZIP10 orthologs from fish and mice. Of note is the absence of positional alignment but the conservation of a mixed charge character N-terminal to the cysteine-flanked core (CFC) domain. ‘G’ and ‘S’ label sites of glycosylation and disulfide linkages, respectively. The α-helix (designated as ‘H’) and β-strand (designated as ‘E’) secondary structural elements known to exist in mouse PrP^C^ are marked. Species abbreviations are: Tr; *Takifugu rubripes*; Dr, *Danio rerio*; and Mm, *Mus musculus*. Dr_ZIP5 was included in the multiple alignment since no Tr ortholog has been identified. (**B**) From left to right, the NMR structure of *Xenopus laevis* PrP (PDB entry 1xu0), a model of *Takifugu rubripes* PrP-1 (residues 28–450), a model of the N-terminal extracellular domain of mouse ZIP5 (residues 116–212), and an overlay depicting the NMR structure of *Xenopus laevis* PrP (green) together with models of Tr_PrP-1 (red) and Mm_ZIP5 (blue) are depicted. In the overlay, stretches of the proteins only shown in one color are in complete overlap amongst the structure and the two models (e.g., parts of α-helices B and C).

A multiple sequence alignment of the predicted globular domain of representative PrP, ZIP5 and ZIP10 sequences from fish (*Danio rerio*, zebrafish; *Takifugu rubripes*, pufferfish) or mammals (*Mus musculus*, mouse) reveals two distinct domains: (i) A segment enriched in amino acids of mixed charge but low positional sequence conservation. In tetrapod PrP^C^, this region contains an α-helix, commonly designated as ‘Helix A’, and a short anti-parallel two-stranded β-sheet. (ii) A region of relatively high sequence similarity demarcated by flanking cysteine residues. The latter domain assembles into ‘Helix B’ and ‘Helix C’ in PrP^C^ of the tetrapod lineage and contains the highly conserved ‘NxS/T’ N-glycan acceptor site known to be physiologically glycosylated in both PrP [32] and a subset of LZTs [12] (**Figure 2A**).

On the basis of a large body of work on tetrapod PrP orthologs [14], [17], the N-terminal half of Tr_PrP-1 is expected to be structurally disordered and refractory to high-resolution structural analyses. In order to gauge the likelihood that the C-terminal half of Tr_PrP-1 (residues 280–450) adopts an α-helical fold similar to that of tetrapod PrPs, we employed the Fold and Function Assignment System (FFAS) server [22] for analysis. The FFAS server utilizes a profile-based sequence alignment and fold recognition algorithm and it predicted that the structure of the C-terminal half of Tr_PrP-1 should indeed resemble the folded domain of other PrP species (**Figure 2B**). The FFAS structural alignment scores were highly significant for many of the PrP structures found in the PDB database with alignment scores in the −40 to −50 range (and a threshold of −9.5 indicating less than 3% false positives). A threading of Tr_PrP-1 (residues 280–450) onto the structure of the African clawed frog (*Xenopus laevis*) PrP structure (XI_PrP; PDB entry 1xu0) gave an alignment score of −48.9 and predicted the presence of the three α-helices, but not the two β-strands (**Figure 2B**). Similarly, the FFAS server's predicted structure of murine ZIP5 (residues 96–212), based on that of Xl*_*PrP (alignment score: −10.9), again indicated the presence of the three α-helices but failed to detect the β-strands (**Figure 2B**). A disulfide bond, corresponding to the one that stabilizes the fold of PrP, is also predicted to exist in Tr_PrP-1 and Mm_ZIP5.

To evaluate the similarity of the Xl*_*PrP structure with the predicted models for Tr_PrP-1 and Mm_ZIP5, we calculated the root-mean-square deviation (RMSD) of the C-alpha atoms via the DaliLite server [23]. The RMSD between the Xl_PrP structure and the Tr_PrP-1 model was 1.2 Å for 98 structurally equivalent residues, while the RMSD between Xl_PrP and the Mm_ZIP5 model was 0.5 Å for 86 structurally equivalent residues. Given the methodology used to generate these models, the RMSD values are not meant to gauge the quality of the models but merely reflect the degree to which the primary structures of Tr_PrP-1 and Mm_ZIP5 are capable of adopting the overall prion protein fold represented by Xl_PrP.

### Bacterial expression of the PrP-like ZIP5 ectodomain leads to protein aggregation

**Figure 3:**
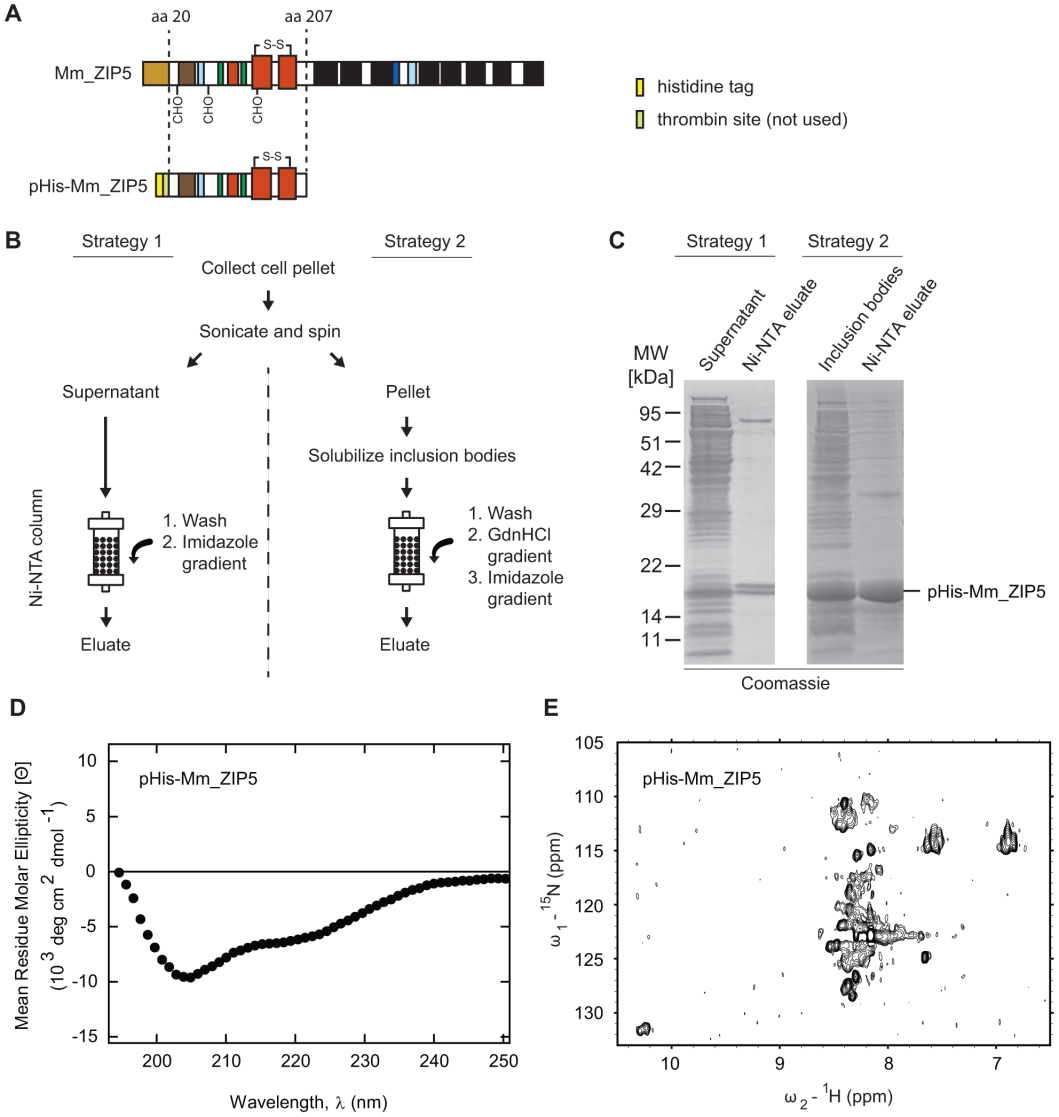
Recombinant PrP-like ectodomain of mouse ZIP5 expressed in bacteria acquires a misfolded conformation. (**A**) Schematic representation of wild-type murine ZIP5 and the derived pHis-Mm_ZIP5 construct used for recombinant expression. Please refer to **Figure 1A** for color codes indicating domains or secondary structural elements. (**B**) Flowchart of alternative strategies for the purification of pHis-Mm_ZIP5 from the soluble fraction of the cellular lysate (Strategy 1) or from insoluble inclusion bodies (Strategy 2). (**C**) Coomassie-stained SDS-PAGE migration profiles of purified pHis-Mm_ZIP5 using the two alternative strategies shown in Panel B. Upon Ni-NTA elution, pHis-ZIP5 can be detected at an apparent molecular weight of approximately 20 kDa, consistent with the molecular mass of the protein. (**D**) CD spectrum of refolded pHis-Mm_ZIP5 purified from inclusion bodies by Strategy 2 indicating the formation of α-helical secondary structure. (**E**). ^15^N-^1^H heteronuclear single quantum correlation (^15^N-^1^H HSQC) spectrum of recombinant purified pHis-ZIP5 showing the amide proton region. The low number of peaks, the clustering of unresolved peaks within the narrow range of 8.2–8.4 ppm in the ^1^H dimension and the broad peak shapes are characteristic of a misfolded protein. Only very flexible regions produced peaks in the spectrum, for example, the C-terminus which maps to 125 ppm ×7.6 ppm.

The goal of obtaining samples of recombinant ZIP5 or ZIP10 for structural analyses was initially based on the bacterial expression of multiple constructs differing in the species of origin and their precise construct boundaries (not shown). For a subset of these, ZIP-coding sequences were inserted into an expression cassette that placed a 10x poly-histidine (pHis) tag and a thrombin protease recognition site N-terminal to the respective ZIP ectodomains. Although the single unpaired cysteine found in the ‘CPALLY’ motif of ZIP5 or ZIP10 was either left intact or mutated to serine, the highly conserved pair of cysteines which demarcate the cysteine-flanked core domain within PrP and the LZTs was left intact. Among more than a dozen expression constructs tested, a construct possessing the entire PrP-like ectodomain of mouse (*Mus musculus*) ZIP5 (Mm_ZIP5) (i.e., lacking the signal peptide and the C-terminal multi-spanning transmembrane domain) exhibited the most promising biochemical characteristics and is described here in some detail (**Figure 3A**). Two alternative strategies were employed for the purification of this pHis-Mm_ZIP5 fragment: (i) a direct purification from the soluble protein fraction following cell disruption by sonication, and (ii) an indirect approach that recovered the majority of the pHis-Mm_ZIP5 protein from insoluble inclusion bodies by a process requiring complete denaturation in 6 M GdnHCl and on-column refolding (**Figure 3B**). In particular, the latter strategy led to highly purified preparations (estimated level of purity >95%) of pHis-Mm_ZIP5 that were soluble over time (**Figure 3C**). Far-ultraviolet circular dichroism (far-UV CD) spectroscopy exhibited characteristics of an α-helical fold but lacked the pronounced minimum at 220 nm observed with proteins exhibiting this secondary structure motif (**Figure 3D**). Moreover, the protein was prone to aggregation in concentrated form (>1 mg/mL) and all attempts to acquire informative well-dispersed two-dimensional ^15^N-^1^H heteronuclear single quantum correlation (^15^N-^1^H HSQC) spectra on pHis-Mm_ZIP5 failed (**Figure 3E**).

### Mammalian expression of the PrP-like ZIP5 ectodomain leads to a soluble homodimeric sample

**Figure 4:**
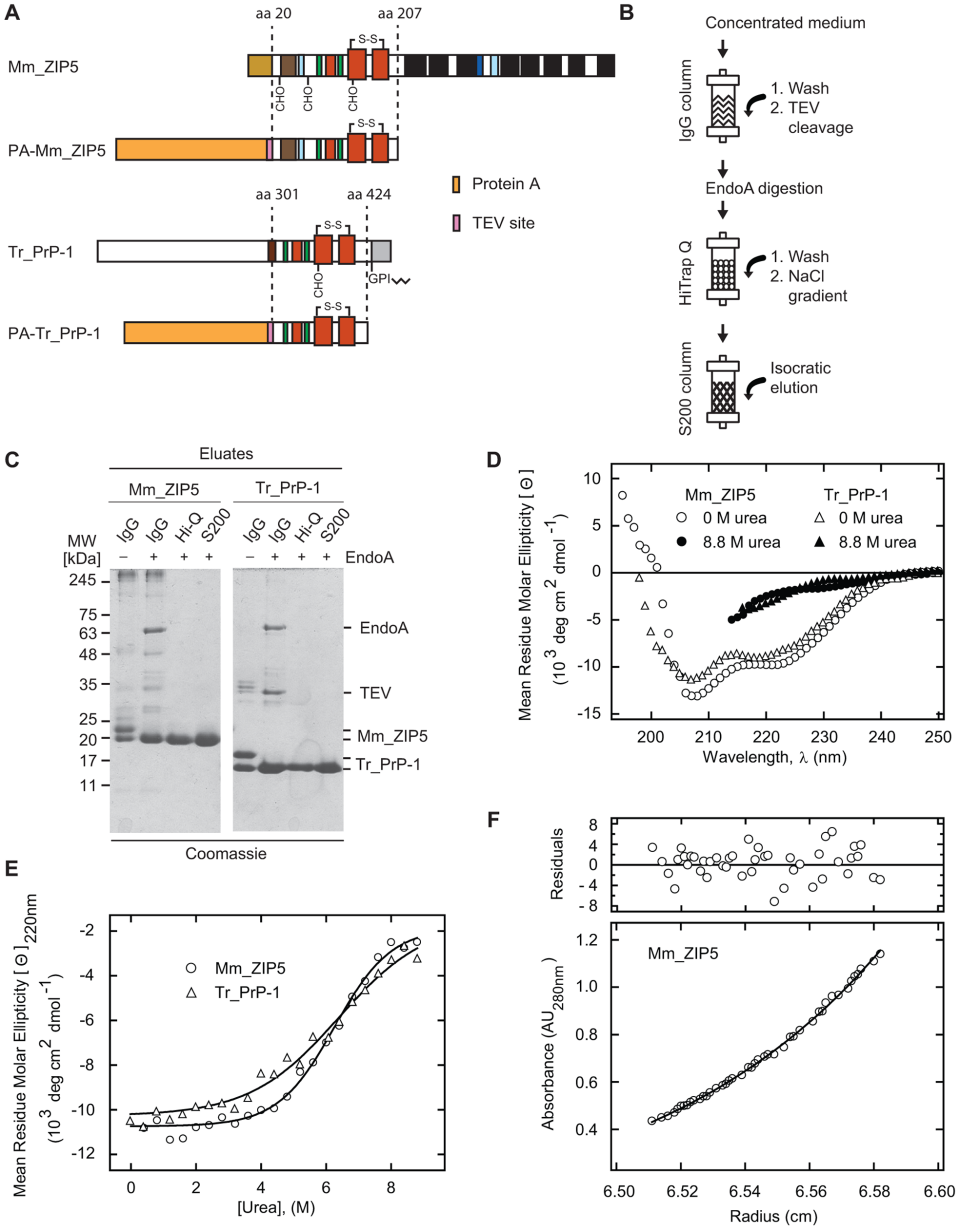
The PrP-like mouse ZIP5 ectodomain purified from mammalian cells gives rise to a homodimeric preparation. (**A**) Schematic depiction of the boundaries and molecular organization of the Protein A (PA)-Mm_ZIP5 (residues 20–207) and PA-Tr_PrP-1 (residues 301–424) expression constructs. Both constructs possess an N-terminal Protein A tag followed by a 15 amino acid linker sequence and a TEV protease recognition site. Please refer to **Figure 1A** for color codes indicating domains or secondary structural elements. (**B**) Strategy employed for the purification of Mm_ZIP5 or Tr_PrP-1 secreted from mammalian cells. (**C**) SDS-PAGE analysis in the course of purification of Mm_ZIP5 and Tr_PrP-1. Of note, deglycosylated Mm_ZIP5 and Tr_PrP-1 migrate with apparent molecular weights of approximately 20 kDa and 15 kDa, respectively. (**D**) Far-UV CD spectra of purified Mm_ZIP5 (open circles) and Tr_PrP-1 (open triangles). CD spectra of urea-denatured Mm_ZIP5 (filled circles) and Tr_PrP-1 (filled triangles) are shown for comparison. (**E**) Urea unfolding titrations of Mm_ZIP5 (open circles) and Tr_PrP-1 (open triangles). Urea denaturation curves (0–9 M urea) were recorded by monitoring the change in the CD signal at 220 nm. (**F**) Sedimentation equilibrium analysis of ZIP5. The lower panel depicts the sedimentation equilibrium profile of 1 mg/mL ZIP5 plotted as A_280 nm_ *vs*. radius measured from the center of the rotor. The data were fitted (solid line) to an ideal single component model with a deduced average molecular mass of 40 kDa. The upper panel shows the distribution of the residuals, i.e., the deviation of each measured data point from the best fit.

To overcome obstacles we and others [16] had encountered during bacterial expression attempts, we moved to a *piggyBac* transposase-based inducible mammalian cell expression system [19] and the human embryonic kidney 293 (HEK-293S GnTĪ) cell line [18]. Experiments focused on the aforementioned Mm_ZIP5 expression construct and a truncated Tr_PrP-1 fragment beginning at alanine-302 and terminating at the predicted glycosylphosphatidylinositol (GPI) anchor attachment site (amino acid 424). Stable bulk cell cultures secreting the N-terminally tagged Protein A fusion proteins (**Figure 4A**) were generated and protein production was scaled-up in roller bottle culture. Subsequent purification (**Figure 4B**) was performed as follows: (i) Immunoglobulin G (IgG) affinity chromatography followed by on-column TEV cleavage to release the PrP-like protein fragments; (ii) Endoglycosidase A (EndoA)-based deglycosylation of N-glycans; and (iii) Q Sepharose anion exchange chromatography. These purification steps were followed by gel filtration chromatography to remove salts and to exchange buffers for downstream biophysical analyses. Western blot analyses showed that the Protein-A-TEV-Mm_ZIP5 fusion protein migrated with a predicted molecular weight (MW) of ∼55 kDa. Following TEV-based cleavage from the IgG column, the expressed Mm_ZIP5 protein fragment was found to migrate on SDS-PAGE as a doublet of 22 kDa and 20 kDa bands that were reduced to a single 20 kDa band after treatment with EndoA (**Figure 4C**). Similarly, the Protein-A-TEV-Tr_PrP-1 fusion protein gave rise to a ∼50 kDa expression product which upon TEV-cleavage and deglycosylation produced a 15 kDa final product.

The far-UV CD spectra of Tr_PrP-1 or Mm_ZIP5 at pH 7.5 were consistent with the purified proteins largely acquiring α-helical folds but they differed consistently from previously reported CD spectra of mammalian PrPs by a less pronounced absorption minimum at 220 nm (**Figure 4D**). CD-based (at 220 nm) urea denaturation experiments on Tr_PrP-1 and Mm_ZIP5 preparations gave rise to sigmoidal plots typically seen for folded proteins (**Figure 4E**). Analytical ultracentrifugation of a highly purified preparation of Mm_ZIP5 indicated that the protein exists as a mono-disperse species with an average molecular mass of approximately 40 kDa, the size expected for a dimer (**Figure 4F**).

### Natural abundance ^13^C-^1^H HMQC analysis of mouse ZIP5 ectodomain

**Figure 5:**
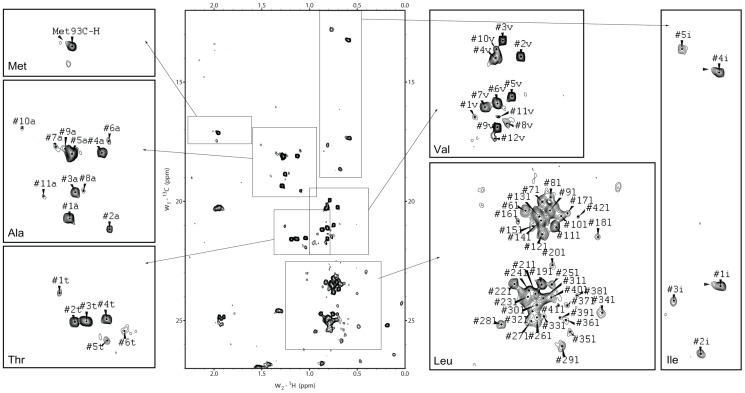
Natural abundance ^13^C-^1^H HMQC spectrum of the deglycosylated ZIP5 ectodomain secreted from mammalian cells. The aliphatic proton region of the ^1^H-^13^C natural abundance heteronuclear multiple quantum correlation (HMQC) proton-carbon correlation spectrum of the PrP-like ectodomain of mouse ZIP5 exhibits many well-resolved and dispersed peaks characteristic of a folded protein. Regions containing methyl group signals that originate from individual types of amino acids are demarcated by rectangular boxes in the main spectrum and are shown at higher resolution in separate subpanels that are labeled with the 3-letter codes of the respective amino acids (predicted composition: 1 Met, 3 Ile, 7 Thr, 8 Val, 15 Ala, 33 Leu). Note that amino acid side-chains of residues containing two methyl groups (Ile, Leu and Val) can give rise to two signals in this type of analysis. Also note the presence of nonsymmetrical peaks (for example, the Met93 and the #1i and #4i isoleucine resonances noted by black arrowheads) indicative of conformational exchange.

To further assess the suitability of the Mm_ZIP5 preparation for structural analyses, a ^13^C-^1^H natural abundance heteronuclear multiple quantum correlation (HMQC) NMR spectrum was acquired (**Figure 5**). Since no ^13^C labeling method was used the signals observed resulted from the ^13^C atoms naturally occurring in the ∼1 mM protein sample. While it is not easy to interpret ^13^C-^1^H HMQC spectra definitively in terms of folding status, particularly in the absence of assignments, the peak dispersion observed may be consistent with a folded state or an equilibrium between a folded and an unfolded state. There are some significantly upfield shifted methyl resonances (<0.5 ppm) outside of the range expected for random coil that are indicative of a folded state hydrophobic core. The variability in intensities, as well as the multiple resonances for the single methionine (note that minor peaks are not always labeled in **Figure 5**), are suggestive of exchange between different conformational states, at least one of which appears to be a folded conformation. Many methyl resonances for the alanine, threonine, isoleucine and valine residues appear as separate peaks. However, a number of methyl resonances of the 33 leucines as well as for the 14 alanines and 8 valines must be significantly overlapping. This might be expected for the leucines due to the presence of multiple instances of two or more consecutive leucine residues as well as the large number of leucines in the protein sequence. Overall, the observation of fewer dispersed peaks than the number of methyl groups in the expressed ZIP5 domain and the variation in peak dispersion and intensities are all suggestive of dynamic exchange consistent with the sampling of a folded state within a larger conformational ensemble, potentially involving dimerization. Optimization of the NMR solution conditions may help to stabilize a single conformation.

## Discussion

The evolutionary relationship between prion genes and the larger family of ZIP zinc transporter genes, and the subsequent biochemical characterization of aspects of the ZIP-PrP interaction, have raised a number of new and interesting questions, two of which this study began to address: (i) do ZIP zinc transporters within the LIV-1 subbranch, known to be most closely related to PrP, possess a similar subcellular distribution as PrP?, and (ii) do PrP and the PrP-like ectodomains present in LZTs acquire a similar fold?

This study suggests that ZIP5 may be a useful target molecule for comparing biochemical features of LZTs and PrP^C^. It established that murine ZIP5 (Slc39a5): (i) is directed by cells to subcellular localizations that strongly overlap with the subcellular location of PrP^C^, (ii) shares with PrP^C^ its uptake from the plasma membrane through Rab5-containing early endosomes, (iii) undergoes N-glycosylation within its PrP-like ectodomain, (iv) is predicted to acquire a fold within its PrP-like ectodomain that, so far, is only known to exist in PrP^C^ and its Doppel protein paralog, and (v) may exist, at least under some conditions, as a dimer. The study further overcame a major obstacle to producing soluble preparations of PrP^C^ orthologs present in fish, thereby paving the way for future high-resolution structural analyses.

It was rationalized that if co-localization was observed, that this might indicate that the respective LZT and PrP^C^ may have retained, throughout evolution, features responsible for their subcellular localization. Further work is required to establish what, if any, functional significance can be assigned to the observed co-localization. The cellular biology of ZIP5 is not well understood. It has been shown that the protein can be directed to basolateral surfaces in a number of polarized cell types [33], [34]. Whereas for PrP^C^, basolateral targeting in Madin-Darby canine kidney cells was observed to depend on its N-glycosylation status and the attachment of a glycosylphosphatidylinositol (GPI) anchor [35], the molecular features that direct the basolateral localization of ZIP5 are not yet known. The expression of ZIP5 is, however, governed by the cellular zinc status, with low levels of available zinc leading to translational repression based on a biology that may be influenced by a predicted stem-loop structure and seed sites for microRNAs found within the 3′-untranslated region of the ZIP5 mRNA [36]. Because N2a neuroblastoma cells do not normally express ZIP5, and its expression in the brain is much lower than, for example, the expression of ZIP10 [37], no claim is made on the basis of data presented here that ZIP5 interacts with PrP^C^ *in vivo* or influences other aspects of its posttranslational cell biology, an interesting question in itself that warrants further investigation.

It could be argued that the boundaries of the constructs selected for the expression of LZT ectodomains may prevent the respective expressed proteins from adopting the proper fold or quaternary state. To minimize these concerns, the N-terminal boundaries of the expression constructs were chosen to either match the predicted N-terminus of the mature protein, following the removal of its respective signal peptide (ZIP5), or to align with the N-terminal boundary of PrP 90–231, one of the most extensively studied PrP fragments [38]. Naturally, the study of N-terminal LZT ectodomains without their respective C-terminal multi-spanning transmembrane domains could preclude interactions that might be important in the formation of their tertiary or quaternary structure. We and others have, however, shown that the N-terminal ectodomains of a subset of LZTs are found following their shedding into the extracellular milieu and, therefore, can be considered physiologically relevant [12], [39].

Based on the data presented in this work alone it cannot be concluded that the dimer observed for the PrP-like ectodomain of ZIP5 secreted from mammalian cells mimics the physiological state of the protein. A number of observations by us and others, however, are consistent with a model that has LZTs assembling into physiological dimers, at least under some conditions. These supporting observations are as follows: (i) the previously reported dimer formation of ZIP13, an LZT for which a thorough biochemical characterization has been undertaken [40]; (ii) the repeated observation of sodium dodecyl sulfate (SDS) stable dimer bands seen for various LZTs during Western blot analyses (see for example [12]); and (iii) the documented ability of members of the PrP subfamily to bind to each other [4], [41], [42]. It has been proposed that induced dimerization may regulate cleavage of PrP^C^ at its α-cleavage site [43]. An interesting twist to the notion that members of the ZIP/PrP protein family can exist as dimers emerged recently from studies that explored the response of PrP to the presence of various divalent cations [44]. Not only did the authors show a Zn^2+^-driven N-terminal to C-terminal tertiary interaction in PrP^C^, but also that the interface within the observed dimer was enriched in residues linked to familial forms of prion disease. Taken together, these data suggested that a divalent cation biology involving zinc may not only modulate dimer formation and, possibly, the function of PrP^C^, but also the conversion of the prion protein.

It is hoped that this work will form the basis for obtaining high-resolution structures of pufferfish PrP and the PrP-like ectodomain of ZIP5, and a comparison with the existing structures of PrPs from other vertebrates. Such information will not only add to the understanding of the relationship between PrP and its ZIP molecular cousins, but may also help to establish if and how PrP might modulate the function of ZIP transporters, a scenario suggested by the initial report of an interaction of PrP^C^ with a subset of ZIPs [5].
